# Current Applications and Future Directions of Technologies Used in Adult Deformity Surgery for Personalized Alignment: A Narrative Review

**DOI:** 10.3390/jpm15100480

**Published:** 2025-10-03

**Authors:** Janet Hsu, Taikhoom M. Dahodwala, Noel O. Akioyamen, Evan Mostafa, Rami Z. AbuQubo, Xiuyi Alexander Yang, Priya K. Singh, Daniel C. Berman, Rafael De la Garza Ramos, Yaroslav Gelfand, Saikiran G. Murthy, Jonathan D. Krystal, Ananth S. Eleswarapu, Mitchell S. Fourman

**Affiliations:** 1 Albert Einstein College of Medicine, Montefiore Einstein, Bronx, NY 10461, USArami.abuqubo@einsteinmed.edu (R.Z.A.); 2 Department of Orthopaedic Surgery, Montefiore Einstein, Bronx, NY 10461, USA; tdahodwala@montefiore.org (T.M.D.); mostafa@montefiore.org (E.M.);; 3 Department of Neurosurgery, Montefiore Einstein, Bronx, NY 10461, USA

**Keywords:** adult deformity surgery, sagittal alignment, spine surgery, patient-specific implants, intra-operative navigation

## Abstract

Patient-specific technologies within the field of adult spinal deformity (ASD) aid surgeons in pre-surgical planning, accurately help identify anatomical landmarks, and can project optimal post-surgical sagittal alignment. This narrative review aims to discuss the current uses of patient-specific technologies in ASD and identify new innovations that may very soon be integrated into patient care. Pre-operatively, machine learning or artificial intelligence helps surgeons to simulate post-operative alignment and provide information for the 3D-printing of pre-contoured rods and patient-specific cages. Intraoperatively, robotic surgery and intraoperative guides allow for more accurate positioning of implants. Implant materials are being developed to allow for better osseointegration and patient outcome monitoring. Despite the significant promise of these technologies, work still needs to be performed to ensure their accuracy, safety, and cost efficacy.

## 1. Introduction

Spinal alignment in the sagittal and coronal planes is essential for balance and overall spinal function. Deviations of the spinal alignment or curvature in the axial, sagittal, or coronal planes from the norm is what define adult spinal deformity (ASD) [1]. Sagittal and coronal plane malalignment can be the result of idiopathic scoliosis or congenital deformations. Other causes of adult spinal malignment are multifactorial, with contributors being osteoporosis, low bone mineral density, and severe degenerative disk disease [2]. Through aging, the disks between vertebrae progressively deteriorate from the weight of gravity, causing an imbalanced load on the vertebrae. Subsequently, vertebral and facet joints will develop arthritic changes due to the loss of disk height, thereby causing ASD, nerve compression, and imbalance [1]. ASD can further predispose patients to future vertebral compression fractures and adjacent segment disease after surgical treatment. Malalignment can cause severe low back and neuropathic pain, disability, and loss of function, including activities of daily living [3]. Patients with a greater degree of sagittal malalignment are associated with an increased risk of complex osteotomies and fixation as well as a worse health-related quality of life (HRQOL) score [4]. An estimated 27.5 million people over the age of 65 nationwide live with ASD [5]. Thanks to technique and technology improvements, ASD surgeries for patients over the age of 75 have become more common and more complex yet less morbid, highlighting the ongoing need to optimize corrective surgery planning to make outcomes more reliably positive [6].

Individual factors, such as BMI, age, comorbidities, ASA grade, and number of operative levels, are predictive of post-operative outcomes such as length of stay and risk of emergency department visits post-surgery [7,8]. Longer hospital stays contribute to increased hospital costs after these procedures [7]. Outcomes after ASD surgery have been associated with the achievement of optimal sagittal alignment parameters [9]. Deviation from ideal alignment increases the risk of disability and junctional kyphosis [10]. Patients with greater pre-operative deformity are at increased risk of not reaching target sagittal alignment parameters, with failure rates of up to 25–30% [11]. Optimal alignment is not “one size fits all,” and age-adjusted sagittal alignment goals have been associated with superior outcomes compared with correction to an “optimal” alignment [12]. Older patients also have medical comorbidities that can make the surgery more challenging [13].

Given the myriad contributors to ASD, including scoliosis, kyphosis, spondylolisthesis, and rotatory subluxation, optimal alignment and patient-reported outcomes following corrective surgery can be significantly enhanced using patient-specific and AI-mediated technologies [3,14]. In a biomechanical study performed by Sardi et al., surgeons on average overbent spine rods by 18.9° without a template [15]. Another report performed by the International Spine Study Group at 13 institutions across the United States found that target sagittal alignment was achieved in only 37.2% of patients without personalized implants [11]. In a comparison study performed by Smith et al., personalized interbody implants allowed for a significantly increased percentage of patients to achieve PI-LL < 5° from the planned target alignment (44.6% vs. 31.5%) [16]. The percentage of patients with PI-LL > 15° from the plan decreased from 30.8% to 15.3% with the usage of personalized interbodies, demonstrating the need for personalization to improve patient outcomes [16]. Such personalized technologies permit a nuanced relationship between surgical factors and patient characteristics such as age, which must be considered to avoid over- or under-correction [12]. The need for individualization has led to the advent of improved pre-operative planning technologies as well as more robust surgical aids and patient-specific implants, significantly improving the reliability and accuracy of deformity correction even in the setting of the challenging anatomy or the multiply operated upon spine [17,18,19].

## 2. Alignment Predictions

Prior to the introduction of intraoperative assistive modalities, postoperative sagittal alignment was significantly dependent upon the experience and talent of the individual surgeon. Early advancements in the prediction of optimal sagittal alignment parameters largely relied on statistical models based on pre-operative radiographs, which has also been expanded to deep learning models [20]. Such statistical models were shown to result in decreased musculoskeletal load and improved postoperative balance in a simulation [21]. Luo et al. has also constructed a prediction model for sagittal alignment prediction after osteotomy in patients with thoracolumbar kyphosis, which were all within 1.3° of actual sacral slope, pelvic tilt, and T1 pelvic angle [22]. Patient-specific statistical modeling found that patients who required revision fusions had initial corrections that more significantly deviated from optimal sagittal alignment [23]. Virtual modeling can also help to predict some of the alignment changes after lumbar surgery and how it contributes to risk for proximal junctional kyphosis [24].

Artificial intelligence (AI) has been gaining traction in use for the prediction of sagittal alignment [25]. Several studies have validated the use of artificial intelligence algorithms in predicting cervical lordosis and alignment from radiographs. AI had a lower error rate than some surgeons [26,27]. One artificial intelligence system (Vertebrai) has shown promise in the measurement of spinal alignment parameters, including kyphosis, spinopelvic tilt, pelvic incidence, cervical lordosis, and lumbar lordosis. Vertebrai had an average 83% accuracy in detecting abnormal values in 0.26 min [28]. SpinePose is another artificial intelligence model that used 761 radiographs for training and validation of sagittal alignment prediction [29]. SpinePose was found to have comparable accuracy to spine surgeons and neuroradiologists [29], and it was also found to be accurate against an external cohort [30]. Beyond alignment prediction, AI and deep learning models are being trained to predict proximal junction kyphosis (PJK) after adult deformity surgery. Brigato et al. found that the AI models predicted PJK with an accuracy rate between 72.5% to 100% [31].

Osteotomy simulation is another avenue in which sagittal alignment prediction is used for surgical planning. Surgimap Spine (Nemaris Inc., New York, NY, USA) is a validated software that is used for ASD surgical planning, especially for osteotomies. Surgimap allows surgeons to simulate post-operative target alignment and calculate how much resection is needed during the osteotomy using preoperative spine radiographs [32]. Surgimap was able to predict postoperative alignment with moderate agreement between Surgimap and actual surgical correction [33], in particular, lumbar lordosis, T1 pelvic angle, and pelvic incidence-lumbar lordosis. However, it was unable to accurately predict postoperative pelvic tilt and sagittal vertical axis [34]. Askyphoplan is another free software that can be used for the prediction of alignment after osteotomies for ankylosing spondylitis, but further studies are needed to validate the accuracy of these software systems [35,36]. Further software development is warranted to continue improving our ability to predict and improve the reliable achievement of sagittal alignment.

## 3. Pedicle Screw Placement

### 3.1. Robotic Surgery

Pedicle screw malposition can pose serious risks to nearby sensitive structures, including the aorta and pleura [37]. Poor screw position can also jeopardize the longevity of the construct. Robotic surgery has been increasingly used in ASD surgery for pedicle screw and S2 Alar-Iliac (S2AI) screw placement. Robotic surgery also has advantages, including decreased radiation exposure and reduced risk of complications [38]. Fan et al. showed that robotic-assisted surgery for pedicle screw placement for adult degenerative scoliosis surgery yielded perfect trajectories 91.3% of the time and clinically acceptable insertion 96.0% of the time. These rates were higher than when a drill guide template or CT intra-operative navigation was used [39]. Chen et al. reported that robotic-assisted scoliosis surgery had a 98.7% clinical accuracy [40], although Karamian et al. did not find any statistical difference in the outcomes of robotic vs. freehand pedicle screw placement during more limited lumbar fusions [41]. In a multicenter clinical study analyzing minimally invasive transforaminal lumbar interbody fusions (TLIF), robotic surgery led to 100% clinically accurate screw placement compared to 92.1% with intraoperative CT-guided navigation [42]. An assessment of robotic S2AI screw placement during deformity surgery found no inaccurate placements or revisions necessary [43]. However, one potential disadvantage of robotic surgery is longer operation times compared to CT-guided navigation and freehand techniques [38,42]. With comparable outcomes to freehand techniques and intraoperative CT-guided navigation, robotic surgery provides the ability to achieve consistent, accurate screw placement regardless of preoperative anatomy [44,45,46,47].

Cost analyses for robotic surgery differ in whether or not robotic-assisted spine surgeries truly reduce the overall cost. Ezeokoli et al. reported that for the initial index surgery, robotic surgery had a higher cost than freehand fluoroscopic techniques due to the large cost of the instruments [48]. Another study found the mean cost of admission for robotic-assisted pedicle screw placement surgery was $69,458 ± $47,910 [49]. Factors that contribute to a higher cost include if the patient has other comorbidities [49]. However, there are also some single-centered studies that found robotic spine surgery to be more cost effective in the long run due to fewer complications, infection rates, revisions, and readmissions [50,51]. One institution estimated saving $608,546 throughout one year after performing 557 robotic cases [51]. More analyses have to be performed to better understand the cost efficacy of robotic surgeries in the short-term and long-term as they become more integrated in clinical practice.

### 3.2. 3D-Printed Patient-Specific Technologies

3D-printed patient-specific guides used in conjunction with the patient’s CT images as part of the MySpine^TM^ (Medacta International SA, Castel San Pietro, Switzerland) software can be used for more accurate pedicle screw placement [52]. Past studies have reported successful and accurate cortical bone screw placement into osteoporotic vertebrae in cadavers, and this has been translated to cervical posterior instrumentation surgery and lumbar spondylolisthesis reduction in actual patients [53,54,55,56]. MySpine^TM^ was shown to have a screw placement accuracy of 96.1% in a clinical trial, greater than the accuracy of freehand techniques (82.9%), suggesting improved safety [57]. In a study with an inexperienced surgeon, cortical bone screw placement accuracy was 91% overall and increased as the surgeon completed more TLIFs [58]. Cool et al. similarly reported 100% screw placement accuracy during thoracolumbar surgery using a superimposed CT-analysis system and 3D-printed patient-specific guides [59]. An additional study reported that 3D-printed pedicle screw jigs resulted in increased screw placement accuracy compared with freehand technique [60]. Decreased complication risk with patient-specific guides has also been suggested [61]. Cortical screw placement with MySpine^TM^ led to a lower creatine kinase level one day and three days post-operatively after TLIFs compared to percutaneous pedicle screw placement, suggesting faster immediate recovery after surgery. Additionally, patients who had cortical screw placement with MySpine^TM^ had decreased perioperative pain compared to percutaneous pedicle screw placement [62]. Faldini et al. also found that patient-specific 3D-printed guides were effective during revision adult spinal deformity surgeries, with a relative accuracy of 94.7% using the Gertzbein-Robbins A+B grading scale [63].

Patient-specific guides and robotic-assisted spine surgery are comparable, and both have their own advantages and limitations. Fan et al. reported that the accuracy of patient-specific guides for screw placement in adult lumbar degenerative disease was 91.79% compared to 90.34% for robotic surgery, which was not statistically different. Robotic surgery led to fewer complications, blood loss, length of stay, and radiation exposure [64]. In a different study by Fan et al. in adult degenerative scoliosis, the accuracy of pedicle screw placement was significantly lower in the patient-specific guide group at 81.3% compared to 91.3% for robotic surgery [39]. However, robotic surgery did lead to longer operation times [39]. Another study comparing 3D navigation systems to robotic surgery found significantly higher accuracy with robotic surgery at the L3, L4, and L5 levels of lumbar surgery but comparable accuracy at S1 [65]. One advantage of patient-specific guides over robotic surgery is the relatively lower cost [66,67]. Furthermore, robotic surgery may be used more for a minimally invasive approach while patient-specific guides may be advantageous in open approaches [64].

3D-printed patient-specific guides have some limitations and potential avenues where they can be applied. Notably, 3D-printed patient-specific guides are custom-made, which means that the design process could potentially take a long time, making these guides unusable in acute situations. Past literature has found that the design and manufacturing process can take anywhere from 4 hours to 3 weeks, depending on the complexity of the case [66]. Furthermore, Yang et al. found that direct patient-specific guides, which required a larger aperture during the operation for direct visualization, had longer design and manufacturing times than indirect patient-specific guides, which used a K-wire for drilling and screw placement [67]. While there has not been much literature regarding cost efficacy of 3D-printed patient-specific guides, it has ranged from $4 to $500 depending on the type of guide. This amount is approximately the same as any other implant [66]. Additionally, 3D models themselves for surgical planning can cost from $175 to $5400 depending on the material of the model [67]. Given these current challenges, AI may help with design and surgical planning to shorten the production time. 3D-printed patient-specific guides may also eventually be applied to minimally invasive surgery [66].

3D-printing has been further expanded to assist with screw placement into “difficult” pedicles. Hypoplastic pedicles (<5 mm) pose multiple challenges during screw placement and stability during thoracolumbar surgery. 3D-printed patient-specific guides for hypoplastic pedicles yielded a complication rate of 9.7%, better than historically reported complication rates [68]. 3D-printed retractors have also been developed to allow for better visualization and placement of pedicle screws during transforaminal lumbar interbody fusions [69].

## 4. Patient-Specific Implants

### 4.1. Patient-Specific Cages

Cages used during lumbar fusion can be made from a variety of materials. Traditional materials included allograft and polyetheretherketone (PEEK), a thermoplastic material that has similar flexibility and durability to bone [70]. Porous titanium cages have been shown to yield greater implant osseointegration than allograft or PEEK [70,71]. Traditional solid titanium cages have largely been replaced by PEEK due to the titanium’s lower flexibility and higher risk of subsidence [72]. Further customization has been developed to allow for cage expansion within the vertebrae [70]. Expandable interbody cages TLIFs had statistically increased foraminal heights post-surgery, improved functional outcomes, and a lower Oswestry Disability Index score [73]. Many other advancements have also been made in the aspects of interbody implants and screws, including porosity of the implant, coating, and other materials [74].

3D-printed titanium cages that can contour precisely to the patient’s anatomy have also been introduced. More precision anatomic contact may lead to improved implant placement and more effective cage expansion, leading to better patient outcomes, fewer complications and revisions, and decreased long-term costs. Time spent planning before the surgery will also decrease, leading to decreased costs for planning the surgery [75]. PEEK and titanium may have different osseointegration properties, but most literature has compared PEEK to 3D-printed titanium implants since PEEK has largely replaced solid titanium cages. In an animal model comparing PEEK, plasma sprayed porous titanium-coated PEEK, and 3D-printed porous titanium cages, the 3D-printed porous titanium cages led to significantly reduced range of motion and increased bone growth within the implant [76]. Compared to PEEK, 3D-printed titanium implants had higher rates of successful lumbar interbody fusion in a clinical trial [77]. Another study found similar or lower revision rates with the use of 3D-printed titanium cages compared to PEEK [78]. A systematic review examined 3D-printed spine surgery implants, where most studies used implants manufactured from titanium (Ti6Al4V) alloy. All papers in the review that reported clinical outcomes demonstrated statistically increased patient-reported outcomes, and the implants were generally found to be safe for implantation [79]. 3D-printed implants provide an advantage because if a patient has complex anatomy, then using generic implants will only increase operating time and risk of complications [79]. Smith et al. reported that in their cohort of 65 patients who had ASD surgery, use of 3D-printed interbody implants compared to stock interbody implants led to a statistically increased number of patients who were within 5 degrees of the planned final alignment after surgery. Fewer patients who received 3D-printed interbody implants had a post-operative alignment >15 degrees [16]. In another study by Calek et al., 97% of interbody levels were fused after one year of surgery using 3D-printed titanium cages for Anterior Lumbar Interbody Fusion (ALIF) and Lateral Lumbar Interbody Fusion (LLIF) surgeries [80].

However, the initial upfront costs of such implants compared with traditional “mass-produced” implants may lead to slow adoption or hesitation. Additional challenges barring the widespread use of 3D-printed cages include the turnaround time required for manufacturing and packaging and the resource demand that can only be met by large industrial companies [81]. A case report described from the Netherlands reported two patients where 3D-printed spinal implants were used. From planning to the insertion of the implant, it took 6 months for the first patient, and the team took the experience from the first patient and spent 6 weeks on the second patient, indicating the potential wide variation in turnaround time [82].

Expandable cages also present with limitations clinically. Compared to static cages, they have a significantly increased risk of cage subsidence, which is when the cage sinks into the vertebra. Chang et al. found that the cage subsidence rate after a TLIF was 19.7% in patients with expandable cages compared to 5.4% in patients with static cages [83]. They may also damage the endplate of vertebrae, which is problematic for patients with osteopenia or osteoporosis [84]. Moreover, bone graft material is not placed in the interbody before expansion, which is different from static cages where bone graft is prefilled. Therefore, packing an expandable interbody with bone fusion material may be challenging [85]. Another large drawback of expandable cages is their increased cost compared to static cages [84,85].

Beyond the development of new biomaterials being used to personalize cages, machine learning has also been applied to predict cage height as well as post-operative spinopelvic parameters. These predictions can guide surgical planning and reduce the risk for complications. The BiLuNet deep learning model as described by Bui et al. was able to predict exact cage height in 54% of the cases and was otherwise able to predict cage height within 1 mm. The model was also able to stratify pelvic incidence-lumbar lordosis with an accuracy of 0.81 [86]. This model is very novel, as there has only been one study testing cage height prediction accuracy from a machine learning algorithm.

### 4.2. Pre-Contoured Rods

Pre-contoured rods applies AI to patient radiographs to measure sagittal alignment parameters and generate a pre-contoured rod based on the patient’s anatomy. Such rods include UNiD^TM^ Adaptive Spinal Intelligence (ASI) (Medtronic; Dublin, Ireland), Expedium^®^ (DePuy Spine, Inc.; Raynham, MA, USA), and ALTALYNE^TM^ Ultra Rods (DePuy Synthes; Raynham, MA, USA), which provide personalized correction during ASD correction. Surgeon experience with the pre-contoured rods has been favorable, with improved postoperative spinopelvic parameters [87,88,89]. Measurement accuracy with UNiD^TM^ was similar to Surgimap [88]. Drexelius et al. found that patients with pre-contoured surgical rods had a higher rate of spinopelvic parameter improvement compared to patients without pre-contoured surgical rods (18% vs. 8.7%). Additionally, patients with pre-contoured surgical rods were less likely to not have corrected parameters (4% vs. 21.3%) [90]. In a comparative study performed by Nasto et al., pre-contoured rods permitted the achievement of all target sagittal alignment parameters except for pelvic tilt. In contrast, patients who received traditional rods had undercorrected pelvic tilt, global tilt, and L1-S1 lordosis. Complication rates were equivalent between the two groups [91]. Faulks et al. also similarly found overall improvement in all sagittal alignment parameters except for pelvic tilt and that patient-reported outcomes improved on average with patient-specific spinal rods [92].

Pre-contoured rods also present with their unique set of potential complications. Pre-contoured rods are usually made of cobalt-chromium, while manually contoured rods are usually made of titanium. The materials inherently give the rod differently properties. For instance, pre-contoured rods are more likely to have plastic deformation, and at larger diameters > 6.0 mm, pre-contoured rods do not have as much of a corrective force compared to manually contoured rods [93]. Additionally, the screw tulips used with the pre-contoured rods may not always match perfectly in size, which could potentially lead to an increased risk of screw dislodgment, hardware failure, and thoracolumbar kyphosis [94]. The risk for proximal junctional kyphosis remains high as reported as 40% in one study [92].

### 4.3. Personalized Interbody Devices

The aprevo^®^ software (Carlsmed, Inc.; Carlsbad, CA, USA) is a new technology that similarly uses the patient’s pre-operative CT images and AI to create 3D-printed personalized interbody devices. The surgeon can use the myaprevo^®^ app to analyze the surgical plan and visualize how the manufactured interbody device fits within the patient’s anatomy. In 2024, the COMPASS registry published the interim results of its prospective study analyzing how aprevo^®^ impacted sagittal alignment after surgery [95]. After a follow-up of at least 24 months, 56.8% of patients who had severe deformity pre-operatively experienced improvement to mild or moderate deformity after use of the interbody device, demonstrating how this device can also help to improve sagittal alignment [95].

## 5. Future Directions

### 5.1. Augmented Reality

Augmented reality is gradually being tested for future use in ASD to further increase precision and patient outcomes [96,97]. When used in the resection of intradural tumors, augmented reality permitted surgeons to feel more comfortable with the anatomy [98]. A feasibility study was performed in cadaver patients, where an augmented reality and artificial intelligence (ARAI)-assisted surgical navigation system was used to overlay images from DICOM radiographs over cadaveric spines to indicate specific anatomical markers that represent landmarks that surgeons might use during a real minimally invasive surgery. ARAI correctly identified all positions at all spinal levels, showing promise for precision if used during minimally invasive ASD [99]. Augmented reality may also serve as a research resource and has been found to have comparable outcomes to cadaveric studies [100]. Augmented reality may be an asset in the future for surgeons to accurately pinpoint patient-specific anatomic landmarks, improving implant accuracy during minimally invasive surgery.

Augmented reality has also slowly been incorporated into clinical practice. Edström et al. compared pedicle screw placement with augmented reality surgical navigation (ARSN) and free-hand. There was increased pedicle screw density with ARSN compared to free-hand, but both groups did not have significant differences in surgery duration or deformity correction [101]. In ASD surgeries requiring spinopelvic screw fixation, the use of augmented reality (Xvision, Augmedics, Heights, IL, USA) compared to free-hand was found to have comparable accuracy rates of 96.5% with augmented reality versus 95.6% by free-hand [102]. In scoliosis surgery, Chang et al. reported that augmented reality allowed for 97.4% screw placement accuracy in the thoracic spine and 100% screw placement accuracy in the lumbar spine [103]. Given that augmented reality is helping with increasing precision, it may continue to be integrated into clinical practice or combined with other robotic technology for continual improvement in continual screw placement.

### 5.2. Biodegradable Implants

Beyond porous titanium and 3D-printed cages, biodegradable implants are gaining traction as a future viable fusion option [70]. Biodegradable cages are largely constructed out of polylactic acid (PLA) or polycaprolactone (PCL). In theory, biodegradable material would be replaced by native bone over time via natural bone remodeling [104]. Therefore, the natural bone remodeling will allow the spine to gradually regain and adjust to its usual load, promoting fusion and decreasing stress shielding, ultimately preventing future development of ASD [105]. Preliminary studies with PLA cages reported inferior outcomes compared with traditional cages due to the lack of strength and durability. PLA cages had lower fusion rates and increased rates of osteolysis [106]. Cages made of PCL had an increased risk of instability over the degradation process, although hybrid formulations have shown more promise than PLA cages. In a sheep model, cages made of PCL mixed with tricalcium phosphate (PCL-TCP) had similar fusion outcomes to titanium implants, with improved osseointegration and comparable strength, stiffness, and range of motion 12 months after surgery [107]. Optimal PCL hybrid formulations are those that replicate properties similar to trabecular bone [108]. A clinical study included 21 patients evaluated cages made of PCL with β-TCP mixed in at a 50:50 ratio, which were 3D-printed for patients who underwent a posterior lumbar interbody fusion (PLIF). Patients had satisfactory outcomes 12 months after the surgery, and 100% had partial cage resorption [109]. It should be noted that 1 cage (excluded from this study) subsided. At 6 months after surgery, the bone fusion rate was 76.2%, and at 12 months after surgery, the bone fusion rate was 95.2% [109]. While biodegradable implants are still being engineered, preliminary data indicate that they may possess mechanical properties that make them potential candidates for use during ASD. Optimal cage material may be patient-specific, with optimal formulations mirroring the patient’s native bone mechanical properties. 3D-printing of these cages may further improve cage personalization [109].

### 5.3. SMART Implants

SMART implants include monitoring devices that can objectively measure a patient’s progress after the surgery. This novel approach to patient care may revolutionize and individualize a patient’s post-operative recovery. SMART devices placed inside spinal interbody fusion cages can monitor a patient’s bone healing by sensing compression forces and potentially predict complications by measuring shear. SMART implants with variable mechanical measurements may be able to alert the surgeon to a potential problem prior to radiographic diagnosis [110]. Similar to a cardiac pacemaker, the spinal SMART implant will behave as a sensor within the implant. It will run on batteries and connect wirelessly to a mobile app, which can track battery life and performance of the implant. Other patient metrics set by the surgeon can be recorded into the mobile app and tracked over time [111]. However, this technology is very novel, and at the time of this review, no published works evaluated newly designed SMART implants.

## 6. Discussion

Patient-specific technologies are rapidly evolving and have many advantages over traditional “off-the-shelf” implants [17]. Statistical modeling and Surgimap can be used to achieve optimal post-operative alignment. Surgeons can simulate outcomes and surgeries to tailor the procedure to the patient’s anatomy [28,32]. 3D-printed implants can achieve target post-operative alignment with comparable complication rates to traditional implants [57,59,60]. In patients with complex anatomy or where revision surgery is required, the combination of biomodelling and patient-specific implants can optimize the patient’s chances of improved functional recovery [112]. Augmented reality can help make surgeons feel more confident in their procedure [98]. Novel biomaterials and SMART materials will improve our ability to monitor patient recovery and fusion.

The present work highlights advancements in patient-specific solutions for ASD. However, substantial gaps still exist in this area. Surgical planning and radiographic alignment prediction models require clinician supervision to maximize their value, leaving some variability in their efficacy [34]. Models incorporating artificial intelligence or machine learning also require large amounts of data and require stringent regulation for patient privacy and security [113]. Because of the need for past data to run these machine learning models, regulation and standardization of these models will be challenging to balance alongside the need for privacy and patient autonomy. Furthermore, artificial intelligence is not 100% accurate and uses certain algorithms and past data to analyze future data. This can introduce systemic biases in the data pool that require someone to validate for accuracy [114]. Given that AI technology often uses patient imaging in its model, a physician may sometimes have to verify whether something in the imaging is actually a malformation or if it is a radiological artifact [66]. Moreover, AI runs on a “black-box” design, which means that physicians cannot always explain how AI obtains inputs and outputs certain information [115]. In the case of an inaccurate output, physicians may not completely know where in the neural network caused an issue for a specific patient, leading to potential difficulties in troubleshooting [115]. Given the easy-to-use features of AI, another issue may be overreliance on AI, decreasing how much safety checks are actually in place [116]. Regulatory practices also present challenges in that every country has different approval processes, and the algorithm and amount of autonomy allowed differ by model [117].

Beyond AI, in the age of value-based medicine, the increased operational costs of personalized implants need to be balanced with improved patient outcomes. More studies and design development are needed to make these technologies accessible and cost efficient to best improve the practicality of these implants [81]. Safety may help demonstrate the value of personalized implants, but these works have not fully evaluated some of the newer biodegradable and SMART materials [81]. Precontoured and 3D-printed rods are largely based around the achievement of sagittal alignment goals. Coronal alignment cannot be measured at present using the UNiD^TM^ system. The ASD literature suggests that patient-reported outcomes may not be completely dependent on post-operative sagittal alignment alone, which likely highlights the value of coronal alignment [118,119,120]. Despite these challenges, we believe that patient-specific implants will become integral to improving the quality of care provided to patients who require ASD.

## 7. Conclusions

Patient-specific technologies, such as artificial intelligence, robotic surgery, and new implant materials, are gaining traction for use in ASD. New patient-specific technologies are helping to improve sagittal alignment and spinopelvic parameters as well as patient-reported outcomes. Further studies and development are still needed to improve the accuracy of surgical planning models as well as the efficacy of new patient-specific implants (Table 1). Patient-specific technology is already a tremendous asset to patient care and surgeons and will continually be integrated into the standard of care.

## Figures and Tables

**Table 1:** Summary of Current and Future Technologies in Adult Spinal Deformity Surgeries.

Technology	Description	Advantages	Future Directions and Areas of Improvement
Artificial Intelligence Models	Artificial intelligence that can measure spinal alignment parameters [25]	Quick speed (under 1 second) in detecting abnormal measurements [28]	Continual improvement in accuracy of measurements
Osteotomy simulation	Used for surgical planning. Can measure alignment from pre-operative radiographs and predict post-operative spinal alignment parameters [32,36]	Simulate osteotomies and provide measurements for amount of resection [34]	Increased accuracy of pelvic tilt and sagittal vertical axis [34]
Robotic Surgery	Used for pedicle screw and S2AI screw placement [38]	Comparable outcomes to freehand and increased accuracy compared to CT intra-operative navigation [40]	Continual improvement in accuracy of screw placement; high cost of index surgery [48]
MySpine^TM^	3D-printed patient-specific guides generated from patient’s CT images [52]	High accuracy cortical screw placement compared to freehand. Decreased perioperative pain and faster recovery after TLIF [63]	Continual training on how to use MySpine^TM^ for more experience, more long-term outcome studies [65]
3D-printed Titanium Cages	Titanium cages 3D-printed based on the patient’s pre-operative images [75]	Tailored to the patient’s complex anatomy, high fusion rates, increased rate of achieving ideal alignment [75]	High cost, time, and manufacturing capacity needed [81]
Pre-Contoured Spinal Rods	Applies AI to patient’s pre-operative radiographs to use alignment measurements and generate pre-contoured rods [89]	Personalized ASD correction [89]	Measurement of coronal alignment, achievement of pelvic tilt alignment [92]
aprevo^®^	Uses AI and patient’s pre-operative CT scans to 3D-print personalized interbody devices [95]	Sagittal alignment improvement [95]	Very new technology that needs more studies to validate alignment improvement and comparison to other devices [95]
Augmented Reality	Augmented reality and artificial intelligence used in combination for navigation during surgery [99]	Accurate screw placement, increased confidence in identification of anatomical landmarks, use in education [99,100]	Needs more studies for safety and how it compares to freehand techniques [102]
Biodegradable Implants	PCL-TCP hybrid implants and other combination of materials that can biodegrade, promoting natural bone remodeling to take place [107]	Properties of the implant may be similar to trabecular bone and allow for osseointegration [105]	Optimization of the combination of materials and testing of safety and efficacy of materials, testing of the strength of materials [106]
SMART Implants	Device can be placed in an interbody fusion to sense compression forces [110]	Can alert to potential complications before indicated on radiographs [110]	Needs more testing for safety and efficacy

## Data Availability

No new data were created or analyzed in this review.

## Abbreviations

The following abbreviations are used in this manuscript:

ASD	Adult Spinal Deformity
HRQOL	Health-related quality of life
AI	Artificial intelligence
PJK	Proximal junction kyphosis
S2AI	S2 Alar-Iliac
TLIF	Transforaminal lumbar interbody fusion
PEEK	Polyetheretherketone
ALIF	Anterior lumbar interbody fusion
LLIF	Lateral lumbar interbody fusion
ASI	Adaptive Spinal Intelligence
ARAI	Augmented reality and artificial intelligence
ARSN	Augmented reality surgical navigation
PLA	Polylactic acid
PCL	Polycaprolactone
PCL-TCP	Polycaprolactone tricalcium phosphate
PLIF	Posterior lumbar interbody fusion
